# Old dilemma: asthma with irreversible airway obstruction or COPD

**DOI:** 10.1007/s00428-015-1824-6

**Published:** 2015-09-14

**Authors:** Fatemeh Fattahi, Judith M. Vonk, Nicole Bulkmans, Ruth Fleischeuer, Annette Gouw, Katrien Grünberg, Thais Mauad, Helmut Popper, Aloisio Felipe-Silva, Bart Vrugt, Joanne L. Wright, Hui-Min Yang, Janwillem W.H. Kocks, Machteld N. Hylkema, Dirkje S. Postma, Wim Timens, Nick H. T. ten Hacken

**Affiliations:** Department of Pulmonology, University of Groningen, University Medical Center Groningen, PO Box 196, 9700 AD Groningen, The Netherlands; Department of Pathology and Medical Biology, University of Groningen, University Medical Center Groningen, Groningen, The Netherlands; Research Institute for Asthma and COPD (GRIAC), University of Groningen, University Medical Center Groningen, Groningen, The Netherlands; Department of Epidemiology, University of Groningen, University Medical Center Groningen, Groningen, The Netherlands; Department of Pathology, Spaarne Gasthuis Haarlem-Zuid, Haarlem, The Netherlands; Department of Pathology, Elisabeth-TweeSteden Ziekenhuis, Tilburg, The Netherlands; Department of Pathology, VU Medical Center, Amsterdam, The Netherlands; Department of Pathology, Sao Paulo University, Sao Paulo, Brazil; Institute of Pathology, Research Unit Molecular Lung and Pleura Pathology, Medical University Graz, Graz, Austria; Department of Surgical Pathology, University of Zurich, Zurich, Switzerland; Department Pathology, University of British Columbia, Vancouver, B.C. Canada; Department of General Practice, University of Groningen, University Medical Center Groningen, Groningen, The Netherlands

**Keywords:** Asthma, COPD, Asthma COPD Overlap Syndrome, Pathology

## Abstract

Older asthmatic patients may develop fixed airway obstruction and clinical signs of chronic obstructive pulmonary disease (COPD). We investigated the added value of pathological evaluation of bronchial biopsies to help differentiate asthma from COPD, taking into account smoking, age, and inhaled corticosteroid (ICS) use. Asthma and COPD patients (24 of each category) were matched for ICS use, age, FEV_1_, and smoking habits. Five pulmonary and five general pathologists examined bronchial biopsies using an interactive website, without knowing patient information. They were asked to diagnose asthma or COPD on biopsy findings in both a pairwise and randomly mixed order of cases during four different phases, with intervals of 4–6 weeks, covering a maximal period of 36 weeks. Clinically concordant diagnoses of asthma or COPD varied between 63 %-73 %, without important differences between pairwise vs randomly mixed examination or between general vs pulmonary pathologists. The highest percentage of concordant diagnoses was in young asthmatic patients without ICS use and in COPD patients with ICS use. In non ICS users with fixed airway obstruction, a COPD diagnosis was favored if abnormal presence of glands, squamous metaplasia, and submucosal infiltrate was present and an asthma diagnosis in case of abnormal presence of goblet cells. In ICS users with fixed airway obstruction, abnormal presence of submucosal infiltrates, basement membrane thickening, eosinophils, and glands was associated with asthma. Histological characteristics in bronchial biopsies are reproducibly recognized by pathologists, yet the differentiation by histopathology between asthma and COPD is difficult without information about ICS use.

**Electronic supplementary material:**

The online version of this article (doi:10.1007/s00428-015-1824-6) contains supplementary material, which is available to authorized users.

## Introduction

Asthma and chronic obstructive pulmonary disease (COPD) are heterogeneous chronic lung diseases, characterized by the presence of airway obstruction and airway inflammation [1, 2]. In asthma, airway obstruction is typically completely or nearly completely reversible [2], while irreversible airway obstruction is typical for COPD [1]. Although both diseases have overlapping clinical features, acknowledged in guidelines [3], they are generally regarded as different disorders; each requiring their own diagnostic and management strategies. For example, for the pharmacological management of asthma, inhaled corticosteroids (ICSs) are the most efficacious drugs currently available [2]. In COPD, inhaled bronchodilators are fundamental for treatment, whereas addition of ICS is only recommended for COPD patients suffering from severe disease (FEV_1_ < 50 % predicted) and/or a history of recurrent exacerbations [1]. In order to determine appropriate treatment, prognosis and follow-up, international guidelines have emphasized the importance of differentiating asthma from COPD.

In general, careful history taking, physical examination, and lung function testing often lead to a clear diagnosis [4]. However, it is frequently difficult if not impossible to achieve an accurate diagnosis of either asthma or COPD in older patients [5--8]. Although asthma generally affects children and young adults, it is not uncommon that asthma starts later in life [9]. In approximately 4 to 8 % of asthmatic cases, the first asthma symptoms are present in late adulthood (late-onset asthma) or even after 65 years of age. With increasing age, a proportion of patients with asthma may develop persistent irreversible airflow limitation, particularly in the presence of risk factors such as smoking [5], blood eosinophilia, chronic mucus hypersecretion, and a low level of FEV_1_ [10]. This type of asthma is clinically indistinguishable from COPD, which the guidelines called ACOS or asthma-COPD overlap syndrome [3], and medical history, physical examination, and lung function tests may become insufficient to distinguish asthma from COPD to allow the most adequate therapy. The lack of a diagnostic standard to identify asthma at older age, together with poor perception of symptoms such as dyspnea, may further hamper the recognition of asthma in the elderly [11].

In case of doubt, clinicians may attempt to achieve a best possible diagnosis of asthma or COPD by taking bronchial biopsies for histopathological examination, although this is not a common practice. It has been suggested that pathological examination of bronchial tissue, taking features such as denudation of the epithelium in asthma and epithelial hyperplasia in COPD into account, might contribute to resolving the diagnostic difficulty. Despite clear morphological differences between asthma and COPD, some morphological characteristics can be found in both diseases (in particular in chronic or severe cases), which impairs their diagnostic value in an individual case [12]. Bourdin et al. demonstrated that the diagnostic value of histological examination of endobronchial biopsies from subjects with asthma or COPD is limited, sensitivity and specificity ranging between 36–48 and 56–79 % respectively [13]. However, the latter study included mostly young, never-smoking, non-steroid-using asthma patients with normal lung function and high bronchodilator reversibility. Furthermore, asthma and COPD patients were not matched for age, airway obstruction, ICS use, and smoking habits. Since these factors modulate histological features of airway inflammation and remodeling, they therefore may have confounded the results.

In the current study, we aimed to identify the most important histopathological features to differentiate between asthma and COPD in bronchial biopsies. We hypothesized that the accuracy of the pathological diagnosis would improve when taking into account modulating factors such as smoking, age, and ICS use.

## Materials and methods

### Patients and matching of biopsies

Biopsies from 24 asthma and 24 matched (see below) COPD patients were included. Subjects were only included when there was no uncertainty in the diagnosis and when subjects met all criteria for either asthma or COPD according to international guidelines [1, 2]. Subjects were selected from several asthma and COPD cohort studies performed in our institute [14--17]. We created three groups (A–C) of asthma patients (*n* = 8 each group), carefully matched with three groups of COPD patients (*n* = 8 each group).

A. The first asthma and COPD groups included subjects who did not use ICS, were >45 years old, had a post-bronchodilator (BD) FEV_1_/FVC <70 %, and had smoked >10 pack-years.
B. The second asthma and COPD groups included subjects with the same criteria, but subjects had used ICS during the last 30 months.
C. The third group included asthma patients without ICS use, and with post-BD FEV_1_ > 90 % predicted, age < 45 years, 0 pack-years smoking, and atopy (Phadiatop > 1.0). This was contrasted with COPD patients without ICS use, with post-BD FEV_1_ < 50 % predicted, age > 45 years, current smoking with >10 pack-years, and without atopy.

Groups A and B included patients with a clinically difficult differential diagnosis between asthma and COPD, whereas control group C included so-called classical cases easy to differentiate.

Table 1 shows the group characteristics (A–C) and Table S1 individual characteristics. Details of the selection process are depicted in the Fig. S1.

**Table 1:** Characteristics of patients with asthma and COPD

	Group A (ICS−)		Group B (ICS+)		Group C (Classical, ICS−)	
Characteristics	Asthma	COPD	Asthma	COPD	Asthma	COPD
Age, years	53 (50–64)	56 (47–63)	61 (54–68)	61.5 (56–72)	29.5 (25–44)	63 (53–64)
Sex, M/F	6M, 2F	5M, 3F	5M, 3F	8M, 0F	4M, 4F	6M, 2F
FEV_1_/FVC,%	64 (48–69)	62 (48–70)	53 (40–66)	53 (36–69)	81 (75–98)	41 (30–47)
FEV_1_, %pred	83 (60–108)	82 (70–106)	83 (43–99)	71 (46–90)	105 (95–122)	45 (41–50)
Pack-years	31 (10–44)	29 (15–43)	20 (12–64)	38 (19–51)	0.0 (0.0–0.0)	32 (21–56)
Current smoking, *n* ex smoking, *n*	8 current, 0 ex	8 current, 0 ex	6 current, 2 ex	6 current, 2 ex	0 current, 0 ex	8 current, 0 ex

Data presented as median (minimum-maximum) except for sex (*M* male, *F* female) and smoking (current, ex smoking). Group A: asthma and COPD patients without ICS use, age > 45 years, post bronchodilator (BD) FEV_1_/FVC <70 %, and >10 pack-years smoking. Group B: asthma and COPD patients with the same criteria, but subjects had to use ICS during last 30 months. Group C: “classical” asthma patients without ICS use, and with post BD FEV_1_ > 90 % predicted, age < 45 years, 0 pack-years smoking, and atopy. Classical asthma was contrasted with classical COPD: no ICS use, post BD FEV_1_ < 50 % predicted, age > 45 years, current smoking with >10 pack-years, and no atopy

Medical records, lung function data, and paraffin-embedded endobronchial biopsies (EBB) were available from all selected patients.

### EBB staining, virtual microscopy, and interactive website

**Fig. 1:**
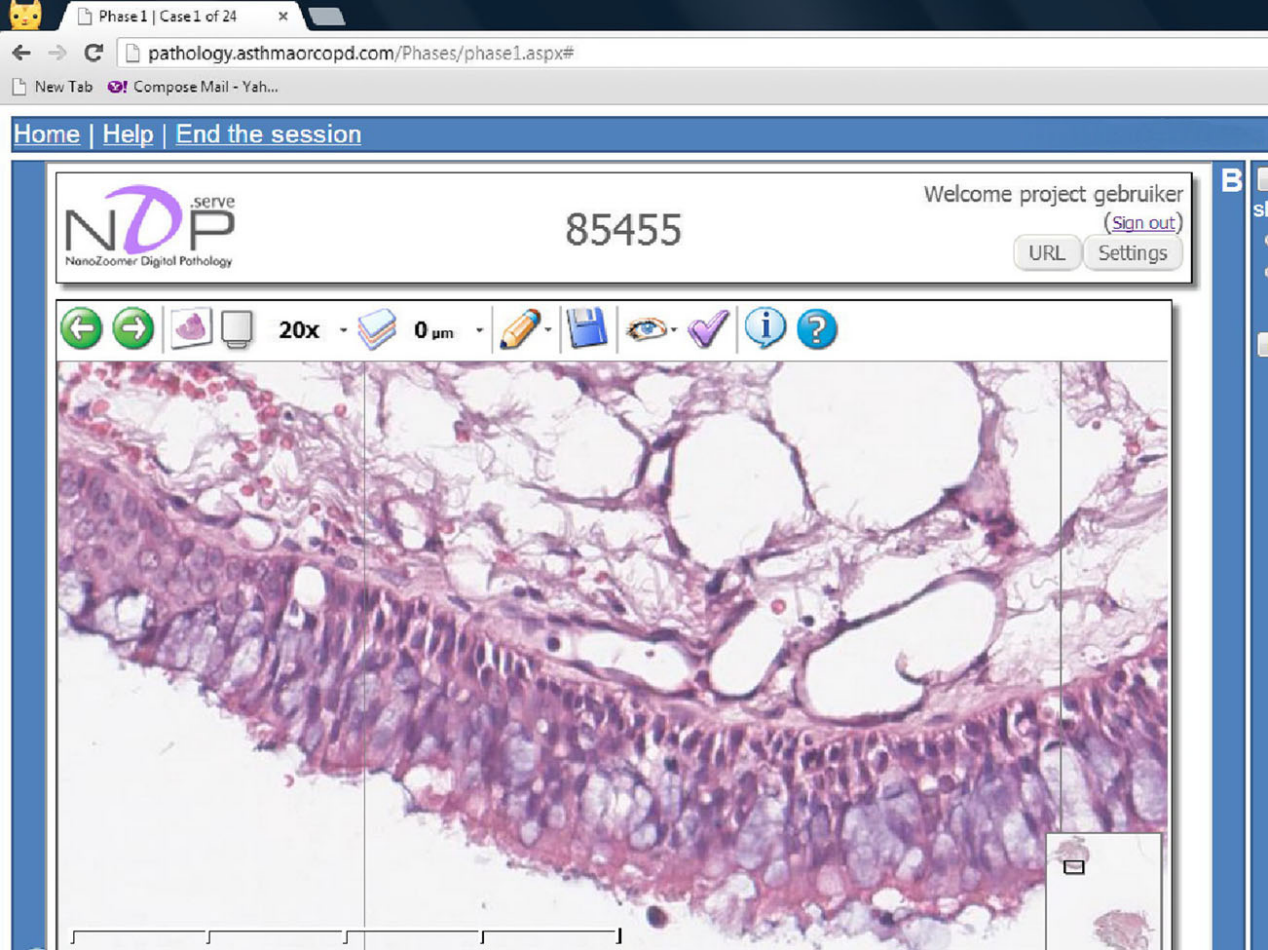
Example slide on interactive website. Screenshot of the interactive website, showing a slide with a representative bronchial biopsy at ×20 magnification. The small image in the lower part of the picture is the overview window, showing the current position and size of the large window. The website allowed to view the slides at different magnifications and to navigate into different areas of the bronchial biopsy like a normal microscope

EBB slides were stained with hematoxylin and eosin (H&E). An experienced pulmonary pathologist (WT) checked the quality of the slides for each patient and selected the best specimen based on size, intactness, and presence of mucosal and submucosal layers. This quality check was performed without knowing the diagnosis or paying attention to the possible diagnosis. Entire biopsies were scanned using Aperio ScanScope Digital Slide Scanner at ×40 magnification. Afterwards, the images were uploaded to a specially designed interactive website, allowing to view the slides at different magnifications and to navigate into different areas of the bronchial biopsy like with a normal microscope (Fig. 1). Quality of our web-based virtual microscopy was checked by an experienced independent pulmonary pathologist who compared all 48 slide images with series of biopsies from the original glass slides by microscope.

### Study protocol

**Fig. 2:**
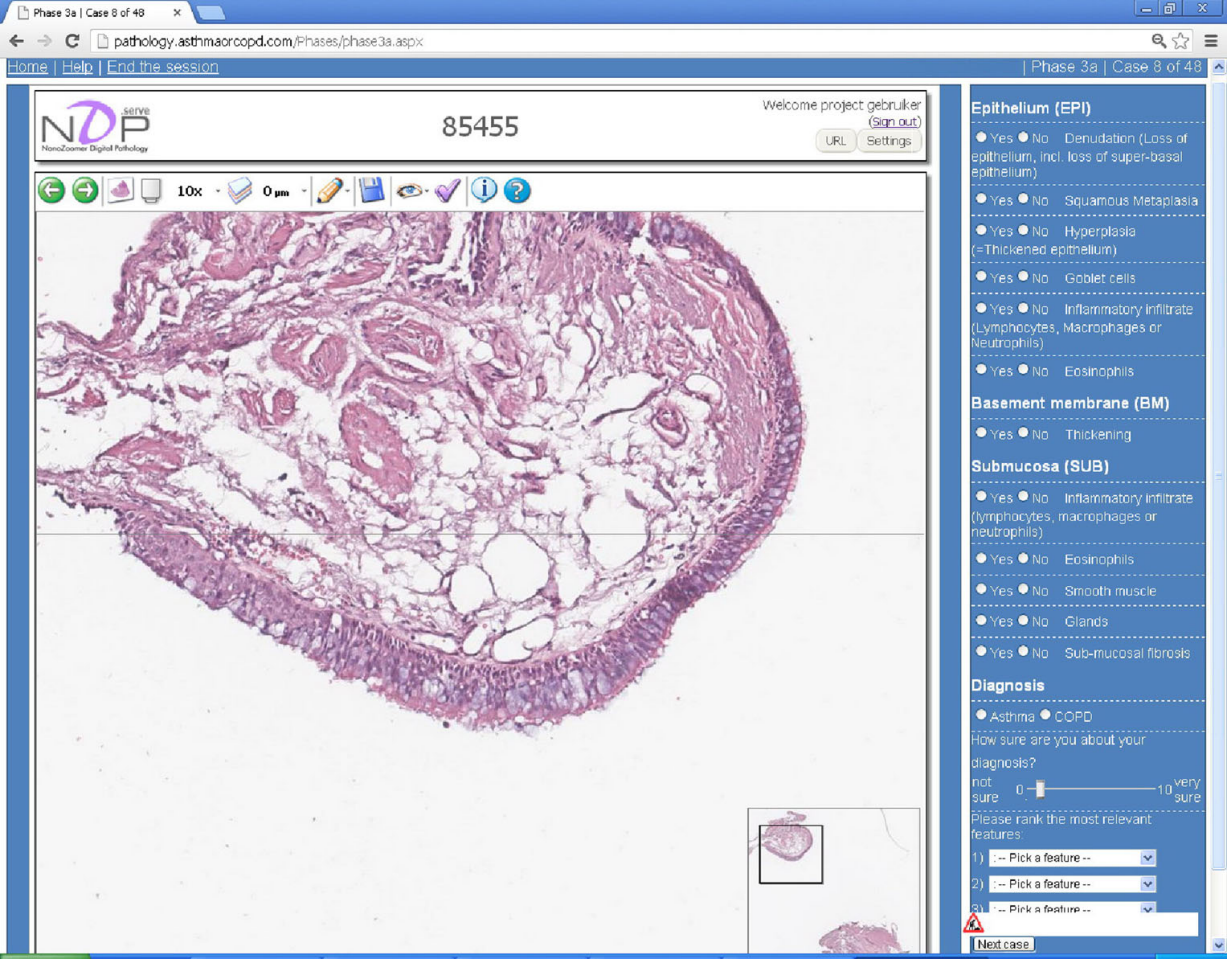
Example slide on interactive website including the list with pathological criteria. Screenshot of the interactive website, showing a slide (phase 3a) with a representative bronchial biopsy at ×10 magnification. The small image in the lower part of the picture is the overview window, showing the current position and size of the large window. At the right side of the slide the pathologists may record the observed abnormal presence of 12 pathological criteria (yes/no), the diagnosis of asthma or COPD, how sure they feel about the diagnosis, and rank the 3 most relevant features

Ten pathologists from different countries (Netherlands, Brazil, Canada, Austria, Switzerland), five of them specialized in pulmonary pathology, participated in the study and used our interactive website following a strict protocol (Table 2). The pathologists were informed about the design of the study but had no clinical information (age, sex, ICS use, smoking, lung function). In phases 1 and 4, the pathologists were offered 48 slides, i.e., 24 pairs of matched asthma and COPD patients, and were asked to indicate per pair which one was asthma and which one COPD. In phases 2 and 3, all 48 slides were offered in a randomly mixed order and the pathologists were asked to choose for either asthma or COPD. In phase 3, the pathologists were additionally asked to indicate the presence or absence of a pathological criterion using the criteria list of Bourdin’s study [13], with small modifications (Box 1). In addition, they were asked how sure they felt about their diagnosis using a 0–10 visual analog scale (VAS) score (0 not sure, 10 very sure) and to rank what they considered the three most relevant features (Fig. 2).

**Table 2:** Design of the study

	Time (weeks)	Examination of slides	Description
Phase 1	2	Pairwise (2 × 24 slides)	Matched asthma and COPD slides were offered pairwise. The pathologists were informed about this and could only opt for asthma or COPD, thus chose for two options per pair. Once chosen, the pathologists were not able to change their choice or go back to slides shown earlier.
Interval	4–6		
Phase 2	4	Randomly mixed (48 slides)	The 48 slides were randomly mixed. The pathologists were informed about this and had to opt for asthma or COPD per slide. Once chosen, the pathologists were not able to change their choice or go back to slides shown earlier.
Interval	4–6		
Phase 3a	2	Randomly mixed + criterion list (48 slides)	Conform phase 2. Additionally, the pathologists were asked per slide to score for the presence or absence of a criterion that drove their diagnosis (Box 1). After scoring the criterion list they had to choose for asthma or COPD and to give their level of certainty (VAS 0–10).
Interval	4–6		
Phase 3b	2	Randomly mixed + criterion list (24 slides)	Conform phase 3a. This phase aimed to test repeatability.
Interval	4–6		
Phase 4	2	Pairwise (2 × 24 slides)	Conform phase 1. This phase aimed to test repeatability (and/or potential learning effects).
	Totally 28–36 weeks

Box 1
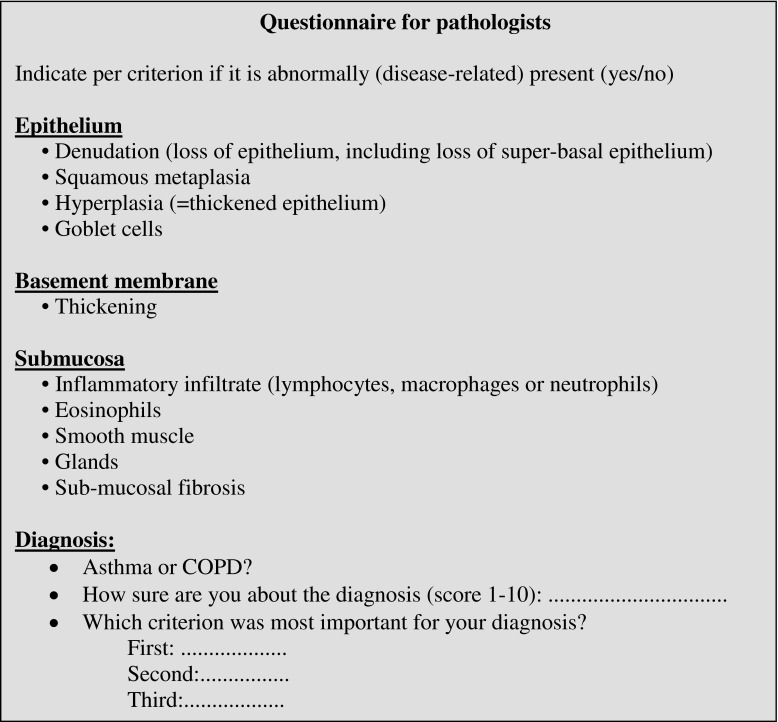


### Statistical analyses

Data from the interactive website were automatically and anonymously saved to an Excel file (MS Excel 2010) and transferred to SPSS software (version 19.0; SPSS Inc., Chicago, IL). The individual data was computed to a concordant or discordant diagnosis of asthma or COPD (i.e., concordant between pathological and clinical diagnosis), and results were expressed as the mean percentage of the concordant diagnosis. In phase 3, the reported presence of each pathological criterion was compared between slides of asthma and COPD using the Mann-Whitney test. Sensitivity, specificity, and accuracy of each pathological criterion for pathologists making a concordant diagnosis of asthma were calculated. A high sensitivity for asthma indicated automatically a high specificity for COPD and vice versa. Pathological criteria with a *p* value <0.2 in the univariate analysis were entered in a logistic regression analysis on the presence of asthma. Pathological criteria which contributed independently to a concordant diagnosis of asthma or COPD were combined to find higher accuracy rates for the concordant diagnosis. Selection of combined criteria was based on the highest Wald value in the regression analyses.

Analyses were performed in the total group of patients, within patient groups (A–C), and between pathologists (general, specialized). Intra- (between phases 3a and 3b, see Box 1) and inter-observer (within phase 3a) agreements were assessed with Cohen’s kappa test and Fleiss’ kappa, respectively. The significance level was set at 0.05.

## Results

### Concordant diagnoses (pathology concordant with clinical diagnosis)

Table 3 shows the percentage of concordant diagnoses per phase, for each disease group (A–C) per pathologist group. The percentages of concordant diagnoses, per pathologist, per phase, are shown in Fig. S2. The percentage of concordant diagnoses of asthma or COPD, per pathologist group, in phase 3a is shown in Table S2. Overall, the highest number of concordant diagnoses was observed in phase 4, particularly by pulmonary pathologists. The highest score for asthma was observed in phase 3a, in the classical asthma/COPD group (C), by pulmonary pathologists, 91.4 %. The highest score for COPD was also observed in phase 3a, in the non-classical asthma/COPD group of ICS users (B), with no difference between pulmonary and general pathologists. Feeling sure about the diagnosis of asthma (in asthma and COPD cases together) was rated on a VAS scale from 1 to 10. In groups A–C, the mean (SD) VAS score for asthma was 5.6 (2.5), 5.2 (2.0), and 6.1 (2.4), respectively. For COPD, this was 5.9 (2.4), 5.8 (2.1), and 5.0 (2.6).

**Table 3:** Percentage of concordant diagnoses in different phases of the study

		Phase 1	Phase 2		Phase 3a		Phase 4
		Pairwise	Asthma	COPD	Asthma	COPD	Pairwise
All pathologists	All	68.7	65.6	62.5	63.5	62.5	72.6
	A (ICS−)	65.0	*67.5*	62.5	*56.3*	*65.0*	63.8
	B (ICS+)	73.8	*52.5*	66.2	*52.5*	*72.5*	76.3
	C (classical)	67.1	*78.6*	58.7	*84.3*	*50.0*	78.6
Lung pathologists	All	71.3	66.1	60.0	70.4	61.7	76.5
	A (ICS−)	62.5	*67.5*	57.5	*67.5*	*57.5*	*62.5*
	B (ICS+)	80.0	*52.5*	65.0	*55.0*	*72.5*	*85.0*
	C (classical)	71.4	*80.0*	57.5	*91.4*	*55.0*	*82.9*
General pathologists	All	66.1	65.2	65.0	56.5	63.3	68.7
	A (ICS−)	67.5	67.5	67.5	*45.0*	*72.5*	65.0
	B (ICS+)	67.5	52.5	67.5	*50.0*	*72.5*	67.5
	C (classical)	62.9	77.1	60.0	*77.1*	*45.0*	74.3

Values are percentage of concordant diagnoses. Italic values: *p* < 0.05 between A, B, and C within 3a phase. Group A: asthma and COPD patients without ICS use, age > 45 years, post bronchodilator (BD) FEV_1_/FVC <70 %, and >10 pack-years smoking. Group B: asthma and COPD patients with the same criteria, but subjects had to use ICS during last 30 months. Group C: “classical” asthma patients without ICS use, and with post BD FEV_1_ > 90 % predicted, age < 45 years, 0 pack-years smoking, and atopy. Classical asthma was contrasted with classical COPD: no ICS use, post BD FEV_1_ < 50 % predicted, age > 45 years, current smoking with >10 pack-years, and no atopy

### Pathological criteria in phase 3

Table 4 shows the reported presence of pathological criteria in the airway wall biopsies in asthma or COPD per disease group (4A–C). Criteria that differed significantly between asthma and COPD were not comparable between the three groups. Eosinophilia was significantly more frequently reported in asthma in groups B + C but not in A (subjects with asthma and COPD in B used ICS, in contrast to subjects in A + C). Submucosal inflammation was significantly more frequently reported in asthma in groups B + C than in group A, yet more frequently in COPD in group A. Table S3 and Box 2 show which pathological criteria significantly contribute to a concordant diagnosis of asthma or COPD in a multiple regression model. Significant criteria present in all groups were goblet cells, inflammatory infiltrate, and glands. Significant criteria present in two groups were eosinophils (group B + C), and in one group squamous metaplasia (group A), BM thickening (group B), hyperplasia (group C), smooth muscle (group C), and submucosal fibrosis (group C). Significant (*p* < 0.05) Wald values of these models are also shown in Table 4.

**Table 4:** Characteristics of the pathological criteria for the diagnosis of asthma or COPD in group A (non-ICS users), group B (ICS users), and group C (classical group)

Criteria	Reported presence (%) in		OR (asthma) (95%CI)	Wald value	Sensitivity (%)	Specificity (%)	Accuracy (%)	Agreement^*^ between pathologists	Repeatability^*^ within pathologist
	Asthma	COPD							
Group A (non-ICS users)									
Denudation	85.0	83.8	1.1 (0.47–2.58)		85.0	16.3	50.6	0.76 (0.77; 0.76)	0.90 (0.95; 0.85)
Squamous Metaplasia	22.5	53.8	0.25 (0.13–0.49)	*7.74*	77.5	53.8	65.6	0.72 (0.77; 0.68)	0.80 (0.90; 0.70)
Hyperplasia	52.5	55.0	0.90 (0.49–1.69)		47.5	55.0	51.2	0.63 (0.73; 0.54)	0.73 (0.75; 0.70)
Goblet cells	60.0	32.5	3.11 (1.63–5.95)	*5.54*	60.0	67.5	63.7	0.85 (0.87; 0.84)	0.85 (0.90; 0.80)
BM thickening	73.8	67.5	1.35 (0.68–2.68)		73.8	32.5	53.1	0.67 (0.69; 0.64)	0.85 (0.85; 0.85)
Inflammatory infiltrate	53.8	72.5	0.44 (0.23–0.85)	*11.84*	46.3	72.5	59.4	0.61 (0.53; 0.69)	0.78 (0.60; 0.95)
Eosinophils	22.5	12.5	2.03 (0.87–4.73)		22.5	87.5	55.0	0.72 (0.64; 0.79)	0.85 (0.75; 0.95)
Smooth muscle	60.0	81.3	0.35 (0.17–0.71)		40.0	81.3	60.6	0.76 (0.78; 0.74)	0.85 (0.80; 0.90)
Glands	23.8	72.5	0.12 (0.06–0.24)	*21.62*	76.3	72.5	74.4	0.85 (0.93; 0.76)	0.90 (1:00; 0.80)
Sub mucosal fibrosis	67.5	58.8	1.46 (0.76–2.78)		32.5	58.8	45.6	0.63 (0.66; 0.60)	0.75 (0.85; 0.65)
Group B (ICS users)									
Denudation	71.3	58.8	1.74 (0.90–3.36)		71.3	41.3	56.2	0.62 (0.61; 0.63)	0.73 (0.65; 0.80)
Squamous metaplasia	33.8	35.0	0.95 (0.49–1.82)		66.3	35.0	50.6	0.80 (0.80; 0.81)	0.85 (0.70; 1.00)
Hyperplasia	51.3	53.8	0.90 (0.49–1.68)		48.8	53.8	51.2	0.67 (0.71; 0.63)	0.90 (0.85; 0.95)
Goblet cells	72.5	60.0	1.76 (0.90–3.41)		72.5	40.0	56.2	0.72 (0.68; 0.76)	0.60 (0.60; 0.60)
BM thickening	57.5	25.0	4.06 (2.07–7.95)	*18.24*	57.5	75.0	66.2	0.71 (0.78; 0.64)	0.75 (0.80; 0.70)
Inflammatory infiltrate	63.8	23.8	5.65 (2.84–11.23)	*19.86*	63.8	76.3	70.0	0.71 (0.73; 0.70)	0.83 (0.80; 0.85)
Eosinophils	30.0	3.8	11.0 (3.16–38.34)	*4.27*	30.0	96.3	63.1	0.85 (0.76; 0.93)	0.83 (0.70; 0.95)
Smooth muscle	66.3	61.3	1.24 (0.65–2.37)		66.3	38.8	52.5	0.77 (0.86; 0.68)	0.80 (0.80; 0.80)
Glands	48.8	33.8	1.87 (0.99–3.53)	*8.88*	48.8	66.3	57.5	0.81 (0.79; 0.83)	0.90 (0.90; 0.90)
Sub mucosal fibrosis	66.3	67.5	0.94 (0.49–1.83)		33.8	67.5	50.6	0.65 (0.64; 0.66)	0.78 (0.85; 0.70)
Group C (classical group)									
Denudation	91.4	80.0	2.67 (0.98–7.25)		91.4	20.0	53.3	0.76 (0.86; 0.68)	0.75 (0.80; 0.70)
Squamous metaplasia	0.0	17.5	0.000 (0.000- -)		100.0	17.5	56.0	0.92 (1.00; 0.86)	0.95 (1.00; 0.90)
Hyperplasia	10.0	61.3	0.07 (0.03–0.17)	*16.95*	90.0	61.3	74.7	0.80 (0.84; 0.77)	0.88 (0.95; 0.80)
Goblet cells	54.3	43.8	1.53 (0.80–2.91)		54.3	56.3	55.3	0.74 (0.73; 0.75)	0.68 (0.70; 0.65)
BM thickening	74.3	71.3	1.17 (0.57–2.40)		74.3	28.8	50.0	0.71 (0.78; 0.66)	0.78 (0.80; 0.75)
Inflammatory infiltrate	91.4	65.0	5.74 (2.21–14.92)	*8.88*	91.4	35.0	61.3	0.72 (0.84; 0.62)	0.73 (0.80; 0.65)
Eosinophils	47.1	20.0	3.57 (1.73–7.34)	*3.97*	47.1	80.0	64.7	0.71 (0.58; 0.83)	0.80 (0.60; 1.00)
Smooth muscle	87.1	77.5	1.97 (0.82–4.72)	*6.34*	87.1	22.5	52.7	0.77 (0.83; 0.71)	0.93 (0.85; 1.00)
Glands	10.0	43.8	0.14 (0.06–0.35)	*8.66*	90.0	43.8	65.3	0.89 (0.89; 0.89)	0.93 (1.00; 0.85)
Sub mucosal fibrosis	14.3	78.8	0.04 (0.02–0.11)	*19.98*	85.7	78.8	82.0	0.72 (0.77; 0.68)	0.78 (0.65; 0.90)

*Repeatability and agreement data presented for asthma and COPD together and separately (asthma; COPD). ORs > 1 indicate a positive association with the presence of asthma; OR < 1 indicate a positive association with the presence of COPD. Sensitivity and specificity for asthma are calculated for the reported presence of variables with OR > 1. Sensitivity and specificity for COPD are calculated for the reported presence of variables with OR < 1. Significant (*p* < 0.05)

Wald values were derived from logistic regression analyses on the concordant diagnosis of asthma and COPD (Table S4)

### Sensitivity, specificity, and accuracy of criteria for a concordant diagnosis

Table 4 shows the sensitivity, specificity, and accuracy for asthma/COPD diagnosis for groups A–C. Pathological criteria with high accuracy differed importantly between groups A–C. In group A (no ICS use), glands, goblet cells, squamous metaplasia, and submucosal infiltrate showed the highest accuracy. In group B (with ICS use), the highest accuracy was provided by submucosal inflammation, basement membrane thickening, eosinophilia, and glands. In group C (classical asthma and COPD), the highest accuracy was shown by submucosal fibrosis, epithelial hyperplasia, eosinophilia, and glands. Combinations of relevant pathological criteria did improve sensitivity or specificity, but in general not accuracy (Table S4).

### Agreement between and repeatability within pathologists

Agreement between pathologists for a concordant diagnosis of asthma or COPD in groups A–C was 0.40, 0.45, and 0.48, respectively. This was 0.33, 0.45, and 0.72 for asthma and 0.60, 0.57, and 0.27 for COPD, respectively. Repeatability within pathologists for a concordant diagnosis in groups A–C was 0.75, 0.78, and 0.80 respectively. This was 0.75, 0.70, and 0.80 for asthma and 0.70, 0.85, and 0.80 for COPD, respectively. Agreement between and repeatability within pathologists for the reported presence of pathological criteria in groups A–C is presented in Table 4. Overall agreement in groups A–C varied between 0.61 and 0.92. Overall repeatability in groups A–C varied between 0.68 and 0.95.

## Discussion

Asthma and COPD are obstructive airway diseases with clear differences in etiology and pathophysiology, yet at older age, they frequently are difficult to discriminate, the currently so-called asthma-COPD overlap syndrome (ACOS) [3]. In the current study, there was no discussion about the original underlying disease, as we had historical data from well-characterized cohort studies. Therefore, we had the opportunity to carefully match bronchial biopsies from 24 asthma and 24 COPD patients taking age, FEV_1_, ICS use, and smoking habits into account. Ten pathologists, not informed about the individual clinical background of the patients, but knowing the study design, were asked to diagnose asthma or COPD. The important outcome of this study is that histological examination of bronchial biopsies alone does not allow differentiating between asthma and COPD. However, as recognition of the specific histological criteria was good, diagnostic value can be expected to improve when selected pathological criteria are applied and adequate clinical information is provided, in which in particular, knowledge about the use of ICS is essential.

In this study, we aimed for high-quality matching of asthma and COPD patients, considering age, smoking, ICS use, and airway obstruction as important modulators of airway inflammation and remodeling. This matching is important because it fits with the original question of the study: whether it is possible for pathologists to discriminate between older asthma patients with fixed airway obstruction and COPD patients, using bronchial biopsies. We designed our study into four phases, gradually increasing the level of difficulty and anticipated that a head-to-head comparison of two paired slides, one from an asthma patient and one from a COPD patient, would help to discriminate between the two diseases. Interestingly, this was not the case. Success rates of scoring of the paired slides were not higher than those of randomly mixed slides. Furthermore, we anticipated that systematic scoring of textbook pathological criteria for asthma or COPD would help to set the correct (=concordant) diagnosis; however, this was not the case.

The overall percentages of a diagnosis of asthma or COPD concordant with the clinical diagnosis were low, taking into account a 50 % concordance by chance. We expected the highest percentage of concordant diagnoses in young asthmatic patients without ICS use and with a normal lung function, assuming that the recognition of the underlying disease is easier when inflammation is not treated with ICS or changed by age- and smoking-related remodeling processes. Indeed, the highest percentage of a concordant diagnosis for asthma in the randomly mixed slides was observed in the group of classical cases (group C, without ICS) and the lowest in the group of asthma subjects who used ICS clinically which are difficult to diagnose (group B). Interestingly, this contrasted with the findings for COPD, where the highest percentage of concordant diagnoses occurred in the group of ICS users. It is not clear at first sight why ICS use reduced the percentage of concordant diagnosis for asthma, yet improved this for COPD. This may well be because corticosteroids reduce eosinophil survival but prolong neutrophil survival; hence, ICS use may have reduced eosinophilia in asthma and increased neutrophilia in COPD [18], eosinophilia being considered a hallmark for asthma and neutrophilia for COPD. This contrasting effect of ICS use on giving a concordant diagnosis of asthma or COPD is an important finding of our study, which—it goes without saying—emphasizes the need for effective transfer of adequate clinical data from clinicians to pathologists.

The reported presence of pathological criteria was significantly different between slides from asthma and COPD patients, in striking contrast with the low percentage of concordant diagnoses of asthma and COPD based on biopsies alone. Additionally, the agreement between the 10 pathologists (inter-observer variability) and repeatability within pathologists (intra-observer variability) was good to excellent for many pathological criteria, independent of a concordant diagnosis. Thus, all expertise to allow a concordant diagnosis of asthma or COPD was present, yet the pathologists appeared to need additional information to be able to apply this expertise successfully, considering the relatively low percentage of concordant diagnoses. A priory, one might expect that the diagnosis of pathologists based upon histology only might be more accurate because it allows integration of all relevant information from all parts of a biopsy. Apparently, this is a difficult task, which is in particular complicated when clinical characteristics like age, smoking, ICS use, and airway obstruction, that affect accuracy and significance of potentially useful pathological criteria, are not known, as shown in the present study.

Next, we tried to identify pathologists with a higher percentage of concordant diagnoses in order to learn and copy their strategy. Beforehand, we expected that pulmonary pathologists would do better than general pathologists. Indeed, at a group level, the mean scores of the pulmonary pathologist group tended to be higher in every phase of the study, and the pathologists with the highest percentages of concordant diagnoses were almost always the pulmonary pathologists. For example, one of the pulmonary pathologists (pathologist B in Table S2) had high accuracy rates, but importantly, in the subgroup of patients who did use ICS (group B), this pathologist had poor results, again demonstrating the importance of knowledge of the clinical background of patients. The pathological criteria that were most frequently ranked as important for the diagnosis by the best scoring pathologists were squamous metaplasia in group A (without ICS use), basement membrane thickening in group B (with ICS use), and submucosal fibrosis in group C (with “classical” asthma and COPD). This is not unexpected as these pathological criteria demonstrated also high accuracy rates at a group level. Finally, we tried to improve accuracy rates by combining pathological criteria that independently associated with the clinical diagnosis of asthma or COPD. Whereas it was not difficult to find combinations with a very high sensitivity or specificity rates, the accuracy rates of these combinations were generally not better than the individual pathological criteria.

We hypothesize that pathologists may improve their ability to differentiate asthma from COPD if they use selected pathological criteria, i.e., those with a high accuracy rate for concordance, provided that the relevant clinical information is known. In non ICS users with fixed airway obstruction the abnormal presence of goblet cells directs towards an asthma diagnosis, whereas glands, squamous metaplasia, and submucosal infiltrate direct towards COPD (Box 2). In ICS users with fixed airway obstruction, the abnormal presence of submucosal infiltrate, basement membrane thickening, eosinophils, and glands direct towards asthma diagnosis. In classical cases, the diagnosis is already known on the basis of clinical characteristics. Nevertheless, the clinical diagnosis can be confirmed by the observation of submucosal infiltrate and eosinophils indicating asthma, whereas submucosal fibrosis, hyperplasia, and glands indicate COPD.

Box 2
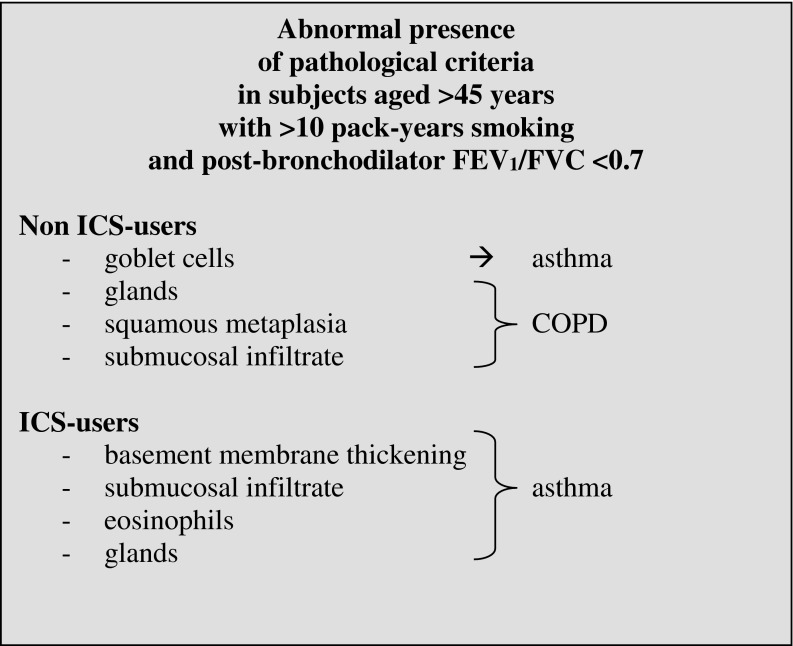


This study has a few limitations. First, one can argue whether or not a physician’s diagnosis of asthma or COPD is an adequate gold standard. Importantly, patient charts of many years back showed that even patients with asthma and fixed airway obstruction at current investigation had reversible airway obstruction at a younger age. After accepting that our physician’s diagnosis is reliable, one might still argue that the selected cases are not the ones that normally would need a pathological examination, because the clinical diagnosis was already established with certainty. Unfortunately, we have no biopsies available to compare cases with certain versus uncertain clinical diagnosis. Our aim however was to establish clues at the microscopical level with strong relation with either asthma or COPD, to be used as clues in difficult biopsies to direct towards a likely diagnosis. Secondly, one could argue that biopsies were not examined by microscope but on screen, which reflects but is not identical to the real-life situation in daily diagnostic practice. This indeed should be validated as is indicated by a recent guideline [19]. Nevertheless, in our approach, all pathologists were exposed to identical images, which improved standardization and allowed pathologists to scroll easily through complete high-resolution slides and varying magnification as in classical microscopy. Third, we did not include slides from healthy controls or from subjects with another lung disease. Consequently, the terms sensitivity and specificity only refer to asthma and COPD, compatible with our study question whether a pathologist is able to give a concordant diagnosis of asthma or COPD. We therefore consider our study design appropriate.

In conclusion, we show that the differentiation between asthma and COPD, based on histopathological examination only of a bronchial biopsy, without adequate clinical information, is difficult. This contrasts with the high percentage of concordant diagnosis observed for a number of reported pathological criteria. We postulate that the diagnostic value is likely to improve when selected pathological criteria (Box 2) are applied and adequate information is provided with respect to the use of ICS. Prospective studies incorporating medical decision techniques may validate algorithms that take these issues into account.

## Electronic supplementary material

ESM 1. (DOCX 220 kb)
