# Supplementary material for: Old dilemma: asthma with irreversible airway obstruction or COPD
Source: Virchows Arch. 2015 Sep 14;467(5):583–93. doi: 10.1007/s00428-015-1824-6 (PMC4656713; doi:10.1007/s00428-015-1824-6)
Supplement: Supplementary file 1 — ESM 1. (DOCX 220 kb) [file 428_2015_1824_MOESM1_ESM.docx]

**Supplement data**

**Old dilemma: Asthma with irreversible airway obstruction or COPD**

Fatemeh Fattahi^1,^^2,3^, Judith M. Vonk^3,4^, Nicole Bulkmans^5^, Ruth Fleischeuer^6^, Annette Gouw^2^, Katrien Grünberg^7^, Thais Mauad^8^, Helmut Popper^9^, Aloisio Felipe-Silva^8^, Bart Vrugt^10^, Joanne L. Wright^11^, Hui-Min Yang^11^, Janwillem W.H. Kocks^3,12^, Machteld N. Hylkema^2,3^, Dirkje S. Postma^1,3^, Wim Timens^2,3*^, Nick H.T. ten Hacken^1,3*^ (* shared last author)

**^1^** University of Groningen, University Medical Center Groningen, Department of Pulmonology, Groningen, the Netherlands,

**^2^** University of Groningen, University Medical Center Groningen, Department of Pathology and Medical Biology, Groningen, the Netherlands.

**^3^** University of Groningen, University Medical Center Groningen, Groningen Research Institute for Asthma and COPD (GRIAC), Groningen, the Netherlands,

**^4^**University of Groningen, University Medical Center Groningen, Department of Epidemiology, Groningen, the Netherlands

^5^ Kennemer Gasthuis, Department of Pathology, Haarlem, the Netherlands

**^6^** Elisabeth-TweeSteden Ziekenhuis, Department of Pathology, Tilburg, the Netherlands

**^7^** VU Medical Center, Department of Pathology, Amsterdam, The Netherlands.

**^8^** Sao Paulo University - Hospital Universitário, Sao Paulo, Brazil

**^9^** Medical University Graz, Institute of Pathology, Research Unit Molecular Lung&Pleura Pathology, Graz, Austria

**^10^** University of Zurich, Department of surgical Pathology, Zurich, Switzerland

**^11^** Dept Pathology, University of British Columbia, Vancouver, B.C. Canada

**^12^** University of Groningen, University Medical Center Groningen, Department of General Practice, Groningen, The Netherlands

**Table S1.** Individual data from asthma patients and pair-matched COPD subjects (group A-C).

|  |  | **Asthma patients** | | | | **COPD patients** | | | | |
| --- | --- | --- | --- | --- | --- | --- | --- | --- | --- | --- |
|  | **Age** | **Sex** | **FEV_1_/FVC**  **(%)** | **FEV_1_**  **(%pred)** | **Pack-**  **years** | **Age** | **Sex** | **FEV_1_/FVC**  **(%)** | **FEV_1_**  **%pred** | **Pack-**  **years** |
| **Group A**  **(ICS-)** | 53 | M | 59.0 | 92.0 | 31 | 54 | M | 58.5 | 92.3 | 30.4 |
|  | 53 | F | 66.3 | 107.8 | 10 | 55 | F | 65.0 | 105.7 | 28 |
|  | 50 | M | 63.3 | 73.2 | 25 | 48 | F | 69.5 | 75.5 | 22 |
|  | 52 | M | 65.3 | 66.5 | 37 | 57 | M | 53.5 | 74.0 | 29.8 |
|  | 57 | F | 63.9 | 93.7 | 15 | 59 | F | 65.4 | 87.7 | 15.3 |
|  | 64 | M | 68.8 | 107.8 | 39 | 47 | M | 67.4 | 102.2 | 28.1 |
|  | 57 | M | 47.6 | 59.8 | 44 | 63 | M | 47.8 | 69.6 | 42.8 |
|  | 52 | M | 60.8 | 67.2 | 30 | 57 | M | 53.5 | 74.0 | 29.8 |
| **Group B**  **(ICS+)** | 68 | M | 39.9 | 56.2 | 12 | 66 | M | 36.3 | 57.8 | 19.3 |
|  | 66 | M | 43.2 | 64.3 | 13 | 72 | M | 41.7 | 65.4 | 20.5 |
|  | 61 | F | 66.1 | 91.1 | 64 | 57 | M | 59.9 | 90.3 | 41.0 |
|  | 61 | M | 50.2 | 42.5 | 34 | 63 | M | 49.7 | 46.2 | 51.0 |
|  | 57 | M | 50.1 | 85.1 | 18 | 56 | M | 50.2 | 70.4 | 28.3 |
|  | 54 | F | 65.6 | 91.3 | 13 | 56 | M | 61.8 | 78.8 | 42.0 |
|  | 59 | M | 55.0 | 81.3 | 34 | 63 | M | 54.8 | 71.5 | 47.0 |
|  | 64 | F | 64.5 | 99.3 | 21 | 60 | M | 68.9 | 79.5 | 35.9 |
| **Group C**  **(Classical)** | 44 | M | 75.4 | 101.5 | 0 | 59 | F | 30.1 | 47.3 | 36.0 |
|  | 38 | M | 81.6 | 107.1 | 0 | 53 | M | 45.1 | 41.1 | 31.2 |
|  | 30 | F | 78.9 | 94.8 | 0 | 64 | F | 47.4 | 49.7 | 32.6 |
|  | 27 | M | 89.6 | 122.0 | 0 | 63 | M | 45.5 | 44.4 | 28.7 |
|  | 25 | F | 97.5 | 107.1 | 0 | 56 | M | 32.7 | 47.7 | 56.2 |
|  | 29 | F | 97.7 | 102.1 | 0 | 63 | M | 41.5 | 46.1 | 30.8 |
|  | 38 | M | 78.7 | 98.8 | 0 | 63 | M | 35. | 40.8 | 55.6 |
|  | 27 | F | 81.2 | 113.3 | 0 | 63 | M | 39.7 | 43.1 | 21.2 |

M: Male, F: Female, ICS: inhaled corticosteroid use. Group A: asthma and COPD patients who did not use ICS, were aged >45 years, had a post bronchodilator (BD) FEV_1_/FVC <70%, and had smoked more than 10 pack-years. Group B: asthma and COPD patients with the same criteria, but subjects had to use ICS during last 30 months. Group C: “classical” asthma patients without ICS use, and with post BD FEV_1_ >90% predicted, age <45 years, zero pack-years smoking, and atopy. Classical asthma was contrasted with “classical” COPD: no ICS use, post BD FEV_1_ <50% predicted, age >45 years, current smoking with >10 pack-years, and no atopy.

**Table S2. Percentage concordant diagnoses per pathologist in phase 3a**

| **Lung**  **pathologists** |  | **Total** | | **Group A** | | **Group B** | | **Group C** | |
| --- | --- | --- | --- | --- | --- | --- | --- | --- | --- |
|  |  | Asthma | COPD | Asthma | COPD | Asthma | COPD | Asthma | COPD |
|  | A | 74 | 54 | 75 | 38 | 50 | 75 | 100 | 50 |
|  | B | 74 | 63 | 100 | 88 | 50 | 25 | 71 | 75 |
|  | C | 70 | 67 | 50 | 63 | 63 | 100 | 100 | 38 |
|  | D | 74 | 54 | 75 | 38 | 50 | 88 | 100 | 38 |
|  | E | 61 | 71 | 38 | 63 | 63 | 75 | 86 | 75 |
|  | *average* | *70* | *62* | *68* | *58* | *55* | *73* | *91* | *55* |
| **General pathologists** |  |  |  |  |  |  |  |  |  |
|  | F | 48 | 67 | 25 | 88 | 50 | 75 | 71 | 38 |
|  | G | 65 | 58 | 38 | 88 | 63 | 75 | 100 | 38 |
|  | H | 52 | 46 | 38 | 38 | 50 | 50 | 71 | 25 |
|  | I | 61 | 75 | 63 | 75 | 38 | 88 | 86 | 63 |
|  | J | 57 | 71 | 63 | 75 | 50 | 75 | 57 | 63 |
|  | *average* | *56* | *63* | *45* | *73* | *50* | *73* | *77* | *45* |

Values are the percentage concordant diagnoses for asthma and COPD per pathologist. Note that some pathologists scored very high for one disease and at the same time very low for the other, showing they favored one diagnosis. Group A: asthma and COPD patients who did not use ICS, were aged >45 years, had a post bronchodilator (BD) FEV_1_/FVC <70%, and had smoked more than 10 pack-years. Group B: asthma and COPD patients with the same criteria, but subjects had to use ICS during last 30 months. Group C: “classical” asthma patients without ICS use, and with post BD FEV_1_ >90% predicted, age <45 years, zero pack-years smoking, and atopy. Classical asthma was contrasted with “classical” COPD: no ICS use, post BD FEV_1_ <50% predicted, age >45 years, current smoking with >10 pack-years, and no atopy.

**Table S3. Multiple logistic regression analysis of reported pathological criteria on the diagnosis of asthma or COPD**

| **Group** | **Criteria** | **B** | **Wald Value** | **P-value** | **Exp (B)=OR** | **Lower (95%CI)** | **Upper (95%CI)** |
| --- | --- | --- | --- | --- | --- | --- | --- |
| Group A  (non-ICS use) | Squamous metaplasia | -1.251 | 7.735 | **0.005** | 0.286 | 0.118 | 0.691 |
|  | Goblet cells | 1.018 | 5.535 | **0.019** | 2.767 | 1.185 | 6.462 |
|  | Inflammatory infiltrate | -1.602 | 11.843 | **0.001** | 0.201 | 0.081 | 0.502 |
|  | Eosinophils | 0.936 | 2.852 | 0.091 | 2.550 | 0.860 | 7.556 |
|  | Smooth muscle | -0.849 | 3.291 | 0.070 | 0.428 | 0.171 | 1.071 |
|  | Glands | -1.960 | 21.617 | **0.000** | 0.141 | 0.062 | 0.322 |
|  |  |  |  |  |  |  |  |
| Group B  (ICS use) | Denudation | 0.664 | 2.312 | 0.128 | 1.942 | 0.826 | 4.567 |
|  | Goblet cells | 0.847 | 3.827 | **0.05** | 2.331 | 0.998 | 5.445 |
|  | BM thickening | 1.988 | 18.241 | **0.000** | 7.304 | 2.933 | 18.189 |
|  | Inflammatory infiltrate | 2.112 | 19.858 | **0.000** | 8.265 | 3.265 | 10.927 |
|  | Eosinophils | 1.573 | 4.267 | **0.039** | 4.819 | 1.084 | 21.433 |
|  | Glands | 1.340 | 8.878 | **0.003** | 3.820 | 1.582 | 9.223 |
|  |  |  |  |  |  |  |  |
| Group C  (Classical group) | Denudation | 1.78 | 3.47 | 0.062 | 5.94 | 0.91 | 38.73 |
|  | Hyperplasia | -3.84 | 16.95 | **0.000** | 0.02 | 0.003 | 0.13 |
|  | Goblet cells | 1.58 | 5.43 | **0.020** | 4.85 | 1.28 | 18.29 |
|  | Inflammatory infiltrate | 2.72 | 8.88 | **0.003** | 15.29 | 2.54 | 91.92 |
|  | Eosinophils | 1.31 | 3.97 | **0.046** | 3.699 | 1.02 | 13.39 |
|  | Smooth muscle | 2.21 | 6.34 | **0.012** | 9.10 | 1.63 | 50.75 |
|  | Glands | -2.63 | 8.65 | **0.003** | 0.07 | 0.01 | 0.42 |
|  | Sub mucosal fibrosis | -3.12 | 19.98 | **0.000** | 0.04 | 0.01 | 0.17 |

Dependent variable: clinical diagnosis for asthma or COPD (+B value: asthma, -B value: COPD).

**Table S4. Examples of combinations of pathological criteria**

| **Group** | **Combinations of pathological criteria**  **presence (+) or absence (-)** | **Sensitivity**  **(%)** | **Specificity**  **(%)** | **Accuracy**  **(%)** |
| --- | --- | --- | --- | --- |
| Group A  (non-ICS use) | Goblet cells + | 60 | 68 | 64 |
|  | Glands – | 76 | 73 | 74 |
|  | Inflammatory infiltrate – | 46 | 73 | 59 |
|  | Goblet cells + and/or glands - | 46 / 90 | 89 / 51 | 68 / 71 |
|  | Goblet cells + and/or inflammatory infiltrate - | 21 / 85 | 95 / 45 | 58 / 65 |
|  | Inflammatory infiltrate – and/or glands - | 33 / 90 | 93 / 53 | 63 / 71 |
|  |  |  |  |  |
| Group B  (ICS use) | BM thickening + | 58 | 75 | 66 |
|  | Inflammatory infiltrate + | 64 | 76 | 70 |
|  | Eosinophils + | 30 | 97 | 63 |
|  | BM thickening + and/or inflammatory infiltrate + | 31 / 90 | 96 / 55 | 64 / 73 |
|  | BM thickening + and/or eosinophils + | 19 / 69 | 100 / 71 | 59 / 70 |
|  | Inflammatory infiltrate + and/or eosinophils + | 27 / 66 | 96 / 76 | 62 / 71 |
|  |  |  |  |  |
| Group C  (Classical group) | Inflammatory infiltrate + | 91 | 35 | 61 |
|  | Submucosal fibrosis – | 86 | 79 | 82 |
|  | Hyperplasia – | 90 | 61 | 75 |
|  | Inflammatory infiltrate + and/or submucosal fibrosis - | 77 / 100 | 85 / 29 | 81 / 62 |
|  | Inflammatory infiltrate + and/or hyperplasia - | 83 / 99 | 79 / 18 | 81 / 82 |
|  | Submucosal fibrosis – and/or hyperplasia- | 76 / 100 | 88 / 53 | 82 / 74 |

Values of sensitivity, specificity, and accuracy are presented for the combination of two pathological criteria; the first value in each cell represents a condition that both criteria are present (the “and” function), the second value represents the condition that at least one of the two criteria is present (the “or” function). Combinations were selected on basis of high Wald values. This table shows that combining two pathological criteria may improve sensitivity or specificity, but in general not accuracy.

**Figure S1. Flowchart of selected biopsies**

This flow chart shows how biopsies were selected on basis of availability of biopsies, age, pack-years, and inhaled corticosteroid (ICS) use. Matching of selected asthma and COPD biopsies into group A, B, and C is described in the methods.


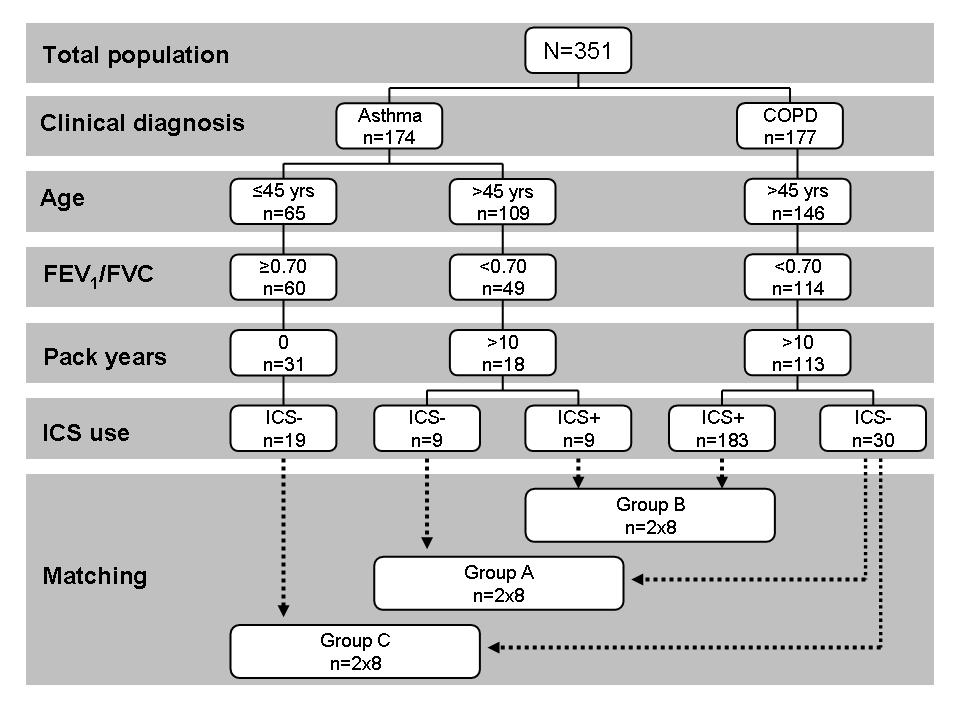


**Figure S2. Concordant answers in different phases**


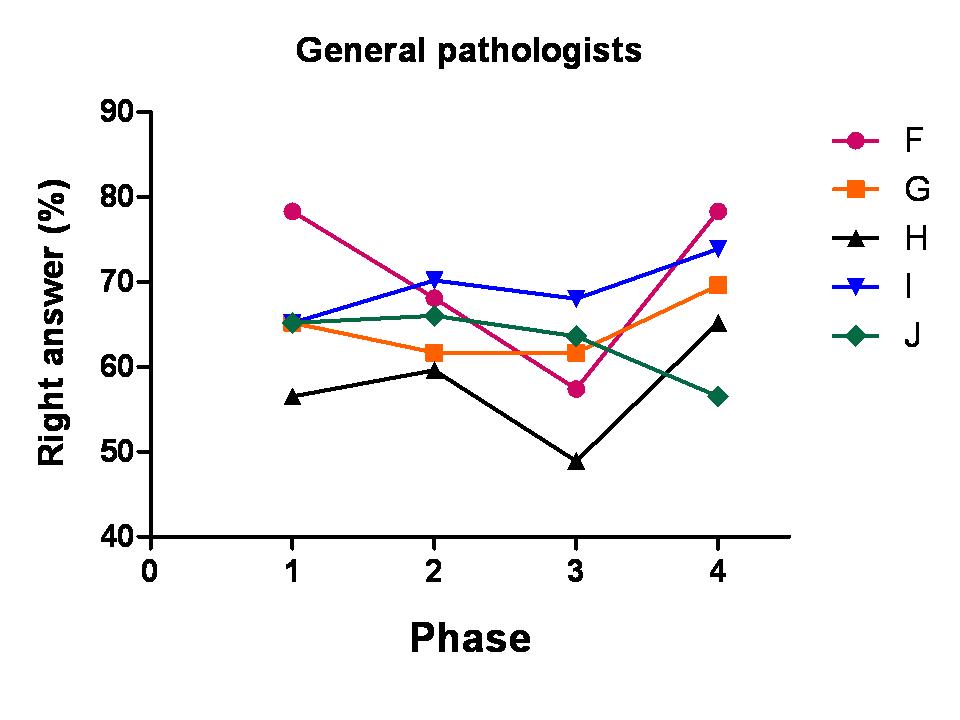

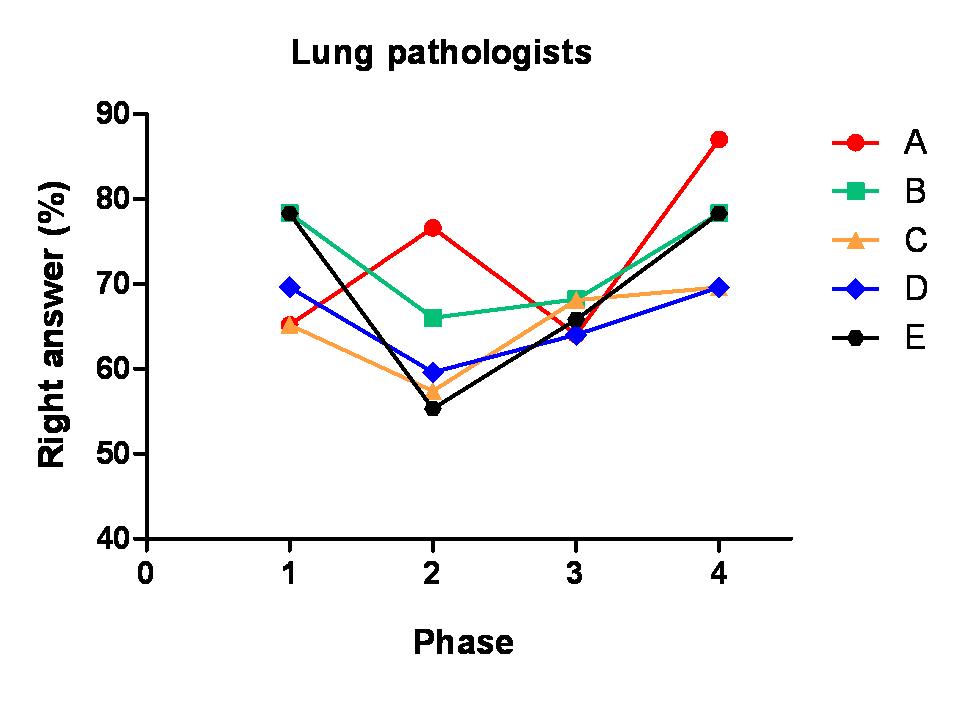


Percentage of concordant answers in the four different phases for the lung pathologists (left panel) and general pathologists (right panel). A-J represent the individual pathologists (see also table S2).

In phase 1 matched asthma and COPD slides were offered pairwise. In phase 2 the slides were randomly mixed. In phase 3 the pathologists were asked per slide to score for the presence or absence of a criterion that drove their diagnosis. In phase 4 matched asthma and COPD slides were offered pairwise again (like phase 1).
